# Model-free measurement of case influence in structural equation modeling

**DOI:** 10.3389/fpsyg.2023.1245863

**Published:** 2024-02-06

**Authors:** Fathima Jaffari, Jennifer Koran

**Affiliations:** ^1^Department of Tests and Measurement, National Center for Assessment, Education and Training Evaluation Commission (ETEC), Riyadh, Saudi Arabia; ^2^Quantitative Methods Program, Southern Illinois University Carbondale, Carbondale, IL, United States

**Keywords:** influence analysis, deletion statistics, Mahalanobis distance, generalized Cook’s distance, Deleted-One-Covariance-Residual (*DOCR*)

## Abstract

In the field of structural equation modeling (SEM), all commonly used case influence measures are model-based measures whose performance are affected by target-model-misspecification-error. This problem casts light on the need to come up with a model-free measure which avoids the misspecification problem. the main purpose of this study is to introduce a model-free case influence measure, the Deleted- One-Covariance-Residual (*DOCR*), and then evaluating its performance compared to that of Mahalanobis distance (MD) and generalized Cook’s distance (*gCD*). The data of this study were simulated under three systematically manipulated conditions: the sample size, the proportion of target cases to non-target cases, and the type of model used to generate the data. The findings suggest that the *DOCR* measure generally performed better than *MD* and *gCD* in identifying the target cases across all simulated conditions. However, the performance of the *DOCR* measure under a small sample size was not satisfactory, and it raised a red flag about the sensitivity of this measure to small sample size. Therefore, researchers and practitioners should only use the *DOCR* measure with a sufficiently large sample size, but not larger than 600.

## 1 Introduction

In structural equation modeling (SEM), normal-distribution-based maximum likelihood (NML) is commonly used as a default estimation method for estimating the parameter values. The NML procedure yields reasonable parameter estimates if the assumption of normality holds in the data distribution. Alternatively, the existence of influential cases in the data might make NML yield biased parameter estimates and affect overall model assessment since these cases could alter the standard error value and the test statistic (Yuan and Bentler, 1998).

One tool that is used for investigating the influence of these cases on the model results is the case influence measures. These measures are built based on the case deletion technique. The case deletion technique is based on the quantification of the impact of the *i*th case by finding the difference between the value of the measure before and after the deletion of the *i*th case to evaluate the impact of this case on the overall model fit. The result obtained from this measure gives information on which case is more influential. In other modeling frameworks, such as OLS regression, there is extensive development and widespread use of case diagnostics for identifying cases, which is not the case with confirmatory factor models, path analysis models, and other models in the SEM framework.

Several regression-based case influence measures have been applied to the SEM field and used with the confirmatory factor models. However, all applied case influence measures are model-based measures that require a theoretical model to be fitted into the data to identify the influential cases. Because case influence measures are model-based, the accuracy of their performance could be impacted by specification errors (Bollen and Arminger, 1991). Since influence measures rely on the structure of the model, they highlight any case that does not fit the model. The determination of one case fits to the model changes depending on the model that has been fitted to the data. Thus, if the model is misspecified, the case influence measure is expected to yield many cases that cause a poor overall model fit. On the contrary, if the case influence measure reflects a few influential cases, it could be expected that the model was correctly specified, and the actual problem of the influential cases existed among the data (Pek and MacCallum, 2011).

The case influence measures that are commonly used in the field of SEM are all model-based measures. Up to this point, no model-free case influence measure has been proposed in the SEM field. Therefore, the main purpose of this study is to avoid the misspecification problem associated with the performance of model-based measures by developing a model-free case influence measure. The proposed Deleted-One-Covariance-Residual (*DOCR*) measure is based on the covariance matrix of the observed data, which allows the *DOCR* to avoid requiring any specific model to fit the data. The *DOCR* uses the deletion technique by comparing the sample covariance matrix that resulted from deleting the *i*th case from the original sample with the sample covariance matrix that resulted from considering all cases in the original sample $\left(s_{i}-s\right).^{2}$ For standardizing the residuals, the residual difference between the two sample covariance matrices, ${\left(s_{i}-s\right)}^{2}$ , is divided by observed variances ( $v_{m}v_{j}p\left(p+1\right))$ . After algebraic arranging, the final formula, as seen in Eq. (1), as follows:


(1) $$\mathrm{DOCR}=\left[\frac{2}{p\left(p+1\right)}\overset{p}{\underset{m=1}{\sum }}\;\overset{m}{\underset{j=1}{\sum }}\;\frac{{\left(S_{i}-S\right)}^{2}}{v_{m}v_{j}}\right]\times 1000$$

Where **S** and **S_i_** are the sample covariance matrices obtained from original and deleted *i*th case samples, respectively. *v_m_* and *v_j_* are the observed variances of each pair of variables in the covariance matrix, and *p* is the number of observed variables. Since the *DOCR* measure would otherwise yield small values that range between 10^−4^ and 10^−5^ for the influence of the cases, the formula of this measure includes multiplying by 1000 to make these values more readable. Our goal is to determine whether the purposed model-free measure *DOCR* precisely identifies the influential cases compared to generalized Cook’s distance (*gCD*) and Mahalanobis distance (*MD*), which are extensively used in multivariate applications to detect outliers. We present the results of two Monte Carlo simulation studies that compared the performance of the proposed measure to the performance of *MD* and *gCD* in identifying the target cases. We hypothesized that the *DOCR* measure would perform better than *MD* and *gCD* in identifying the target cases across variations in sample size, proportion of target cases, and model specifications.

### 1.1 Background

In SEM, the case influence measures aim to evaluate the degree of the model fit at the person level; stated differently, they aim to identify unusual cases under the model (Reise and Widaman, 1999). Corresponding to regression, the following factor analysis model as seen in Eq. (2) is considered a latent predictor’s multivariate regression model:


(2) $$X_{i}=\mu +\Lambda f_{i}+e_{i}$$

Where **μ** is a population mean vector, **Ʌ** is a *p × q* factor loadings matrix, **f_i_** is a vector of q-variate latent factors, and **e_i_** is a vector of measurement errors. Based on this factor model, Yuan and Zhong (2008) stated that the cases with large absolute values of measurement error (**e_i_**) are termed *outliers*, disregarding the values of the factor scores (**f_i_**). The cases with extreme absolute values on the exogenous latent variables’ factor scores are termed *leverage cases*. Leverage cases with a small magnitude of measurement errors (**e_i_**) are considered *Good Leverage Cases*, while leverage cases with a large magnitude of measurement errors (**e_i_**) are considered *Bad Leverage Cases*. In SEM, unusual cases with large $e_{i}$ are considered influential on both the model fit and the parameters since they cause a large change in the off-diagonal elements of **S** (sample covariance matrix). Case influence measures use the deletion technique to quantify the influence of these cases by comparing the value of the statistic before and after the deletion of the *i*th case from the data. Most of these measures have been proposed and developed in the regression field (Belsley et al., 1980; Cook and Weisberg, 1982). However, some of these statistics have been applied to the SEM field to identify the influential cases and quantify their influence on the model findings.

One of the deletion measures that have been applied to SEM is *gCD*. *gCD* is a model-based measure that is used to quantify the influence of the unusual case on the parameter estimates. This measure is a generalized version of Cook’s distance (Cook, 1977, 1986). Atkinson (1981) modified Cook’s distance for influential case detection by adding the values of the parameter estimates after deleting the *i*th case and controlling for the sample size effect. Then, Lee and Wang (1996) used the generalized least square function to generalize Cook’s distance measure to the SEM application.

*gCD* has been introduced and used in some studies (Zhao and Lee, 1998; Pek and MacCallum, 2011) to examine the case influence on a set of *l* parameters on a set of *l* parameters, as seen in Eq. (3).


(3) $$gCD_{i}={\left(\hat{\theta }-{\hat{\theta }}_{i}\right)}^{\prime }\;{\left[\hat{VAR}\left({\hat{\theta }}_{i}\;\right)\right]}^{-1}\;\left(\hat{\theta }-{\hat{\theta }}_{i}\right)$$

Where $\hat{\theta }$ and $\hat{\theta }$ ***_i_*** are vectors of parameter estimates that are calculated from all cases in the original sample and the sample with the *i*th case deleted, respectively. The $\hat{VAR}$ ( $\hat{\theta }$ ***_i_***) is the estimated asymptotic covariance matrix of the parameter estimates calculated from the sample with the *i*th case deleted. Assuming that *k* is the full set of the model parameters and *l* is the number of the desirable subset of the model parameters, one can calculate *gCD* for any subset of parameters *l* instead of the full set of model parameters *k*.

Given the *gCD* quadratic form, the lower bound of *gCD* is equal to zero, which means that this statistic always takes positive values, and that makes *gCD* give us information on the level of change rather than the direction of the change on the model parameters. Thus, a small amount of *gCD* means that a small change in the *l* subset of parameter estimates is associated with the exclusion of the *i*th case from the sample. On the other hand, a large amount of *gCD* means that a large change in the *l* subset of parameter estimates is associated with the exclusion of the *i*th case from the sample.

To obtain information about the direction of change in an individual parameter, the scaled difference $\left(\Delta \hat{\theta }ji\right)$ is used for this specific purpose (Zhao and Lee, 1998; Pek and MacCallum, 2011) as seen in Eq. (4).


(4) $$\Delta {\hat{\theta }}_{ji}=\frac{{\hat{\theta }}_{j}-{\hat{\theta }}_{j\left(i\right)}}{\sqrt{\hat{VAR}}\left(\;{\hat{\theta }}_{j\left(i\right)}\right)\;}$$

Where $\hat{\theta }$*_j_* and $\hat{\theta }$*_j_*(*_i_*) are the parameter estimates obtained from the original and deleted *i*th samples, respectively. Positive values of difference indicate that small change is associated with the exclusion of the *i*th case and vice versa.

Other case diagnostic measures have been developed for latent variable models (Pek and MacCallum, 2011; Sterba and Pek, 2012). However, these three measures (i.e., *LD*,$\Delta \chi^{2}$, and *gCD*) are currently the most readily available due to their inclusion in the R package influence. SEM (Pastore and Altoé, 2022).

Due to the slow development of case influence measures in SEM, *MD* is routinely used in multivariate applications to detect unusual cases. *MD*, as seen in Eq. (5), is the distance between the *i*th case and the remaining cases while accounting for the correlation in the data (Mahalanobis, 1936). Some studies used the main and derived versions of this test mainly for detecting the potential multivariate outliers and leveraged cases (Pek and MacCallum, 2011; Yuan and Zhang, 2012).


(5) $$MD^{2}=\left(Y_{i}-\bar{Y}\right)\;C^{-1}{\left(Y_{i}-\bar{Y}\right)}^{\prime }$$

(6) $$C=\frac{1}{n-1}\;{\left(Y_{c}\right)}^{\prime }\left(Y_{c}\right)$$

Where **Y** is an *N × p* data matrix containing *N* cases on *p* variables, $Y_{i}$ is a 1 × *p* vector of *p* variables for the *ith* case, $Y_{c}=Y-\bar{Y}$ is the column-centered data matrix, $\bar{Y}$ is an *N × p* matrix of the column means, _f_ and and **C**, as seen in Eq. (6), is the variance–covariance matrix (De Maesschalck et al., 2000, p.2). *MD*^2^ distributes as a central chi-square distribution with degrees of freedom (*df*) equal to the number of variables. A significantly low value of p of high *MD_i_*^2^ in the corresponding $\chi^{2}$ (*df*) means that the *i*th case is a potential outlier (Kline, 2016, p. 73).

However, *MD* is a model-free measure of outlying status rather than case influence, and it is generally used in multivariate applications to detect outliers (Mahalanobis, 1936). In practice, some researchers use *MD* to identify the outliers and delete them prior to fitting the model to the data. The problem with this practice is that influential cases could be outlying cases (i.e., outliers), but not all outlying cases are influential. That is, some outlying cases are not regression outliers because they do not deviate from the linear pattern of the data, so they are considered good cases since their inclusion in the estimation process could lead to a better overall model fit and precise parameter estimates (Rousseeuw and van Zomeren, 1990). Based on this fact, Pek and MacCallum (2011) recommended against using such practice since the removal of good cases, because *MD* identifies them as outlying cases, might lead to worsening the overall model fit. Thus, this practice sheds light on the limitations of using *MD* in the case influence analysis to identify influential cases. On the contrary, model-based measures demand to fit a theoretical model to the data for quantifying the impact that each case exerts on the findings of modeling. The latter measures consider the structure of the model, and their values change as the model structure and set of independent variables change (Belsley et al., 1980).

The purpose of this study is to introduce a model-free case influence measurement that overcomes the problem of specification error and the limitations of using an outlying status measure (i.e., *MD*) in identifying the influential cases. This proposed measure is compared to *MD* and *gCD* to evaluate its ability to identify target cases under a variety of systematically manipulated conditions while accounting for sampling variability using Monte Carlo simulation.

## 2 Methods

### 2.1 Data generation

#### 2.1.1 Simulation study 1

The data for this simulation study were generated under a population confirmatory factor analysis (CFA) model with two factors and three indicators per factor. For scaling the factors, the unit variance identification method was used. Target cases were generated from a $N\left(0,2.25I_{6}\right)$ distribution (c.f., Lee and Wang, 1996), where $I_{6}$ is a 6 × 6 identity matrix. Non-target cases were generated using the common factor model $N\left(0,\Sigma \right)$ , where $\Sigma =\Lambda \Phi \Lambda^{\prime }+\Psi$ is the 6 × 6 population covariance matrix, Λ is the loading matrix with $\Lambda^{\prime }=\left[\begin{array}{ccc} 0.8 & 0.8 & 0.8\\ 0 & 0 & 0 \end{array}\;\begin{array}{ccc} 0 & 0 & 0\\ 0.5 & 0.5 & 0.5 \end{array}\right]$ , $\Phi =\left[\begin{array}{cc} 1.0 & 0.6\\ 0.6 & 1.0 \end{array}\right]$ is the factor correlation matrix, and $\Psi =\mathrm{diag}\left\{0.36,0.36,0.36,0.75,0.75,0.75\right\}$ is the 6 × 6 diagonal matrix of unique variances.

#### 2.1.2 Simulation study 2

The data for this simulation study were generated under a population path model with five observed variables. Data sets were simulated with target cases from $a\;N\left(0,6.49I_{5}\right)$ distribution, where $I_{5}$ is the 5 × 5 identity matrix, and 6.49 was the result of multiplying the largest variance in the diagonal of the covariance matrix of the data by 4 following the same process of generating the target cases used within the first simulation study (c.f., Lee and Wang, 1996). Non-target cases were generated using the population path model from $a\;N\left(0,\Sigma \right)$ , where

**Y** = Γ **X** + **BY**+ ζ , Γ = $\left[\begin{array}{cc} 0.7 & 0.6\\ 0 & 0.6\\ 0 & 0 \end{array}\right]$ , **B** = $\left[\begin{array}{ccc} 0 & 0 & 0\\ 0 & 0 & 0\\ 0.5 & 0.6 & 0 \end{array}\right]$ ,

Σ is the v × v population covariance matrix, Γ is a parameter matrix of the direct effect of exogenous variables on the endogenous variables, **B** is the parameter matrix of the direct effect of endogenous variables on each other, and ζ is the matrix of the disturbances.

### 2.2 Case diagnostics

**Figure 1:**
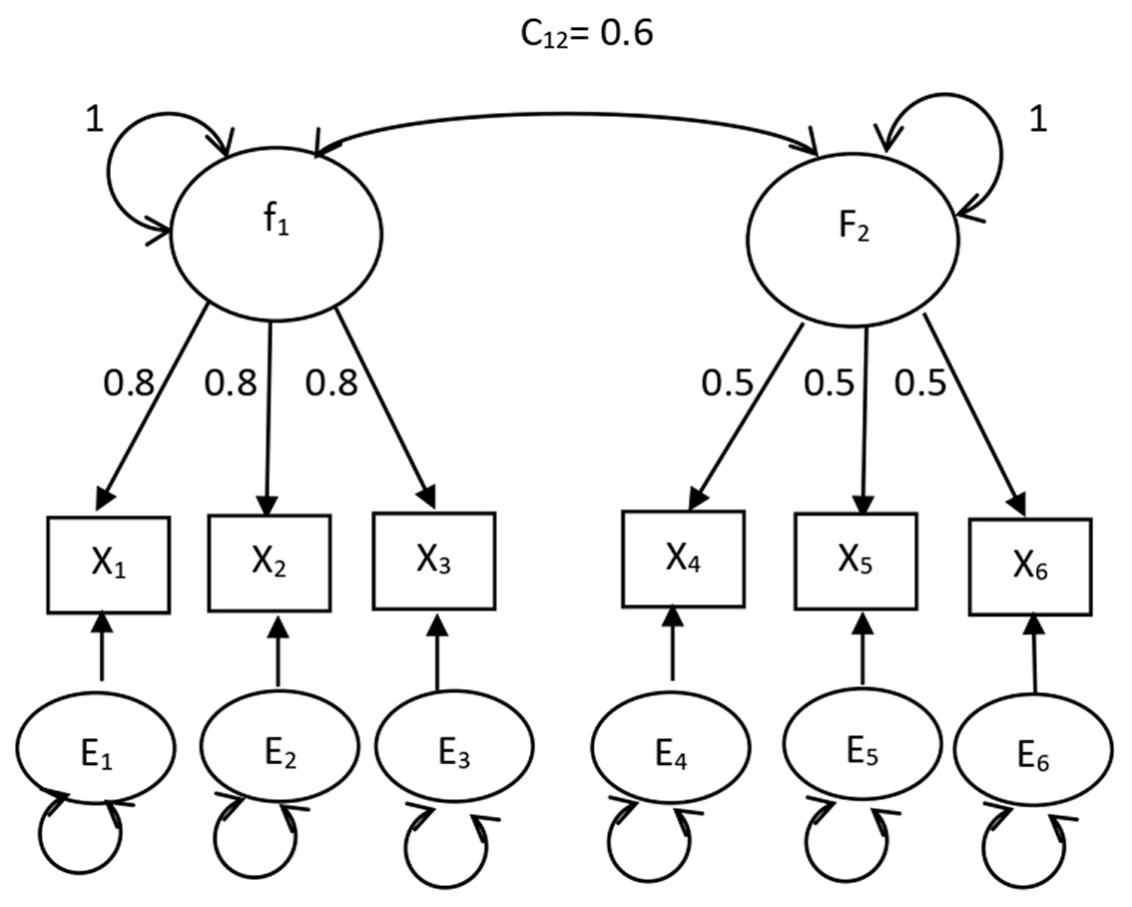
The correctly specified common factor model.

**Figure 2:**
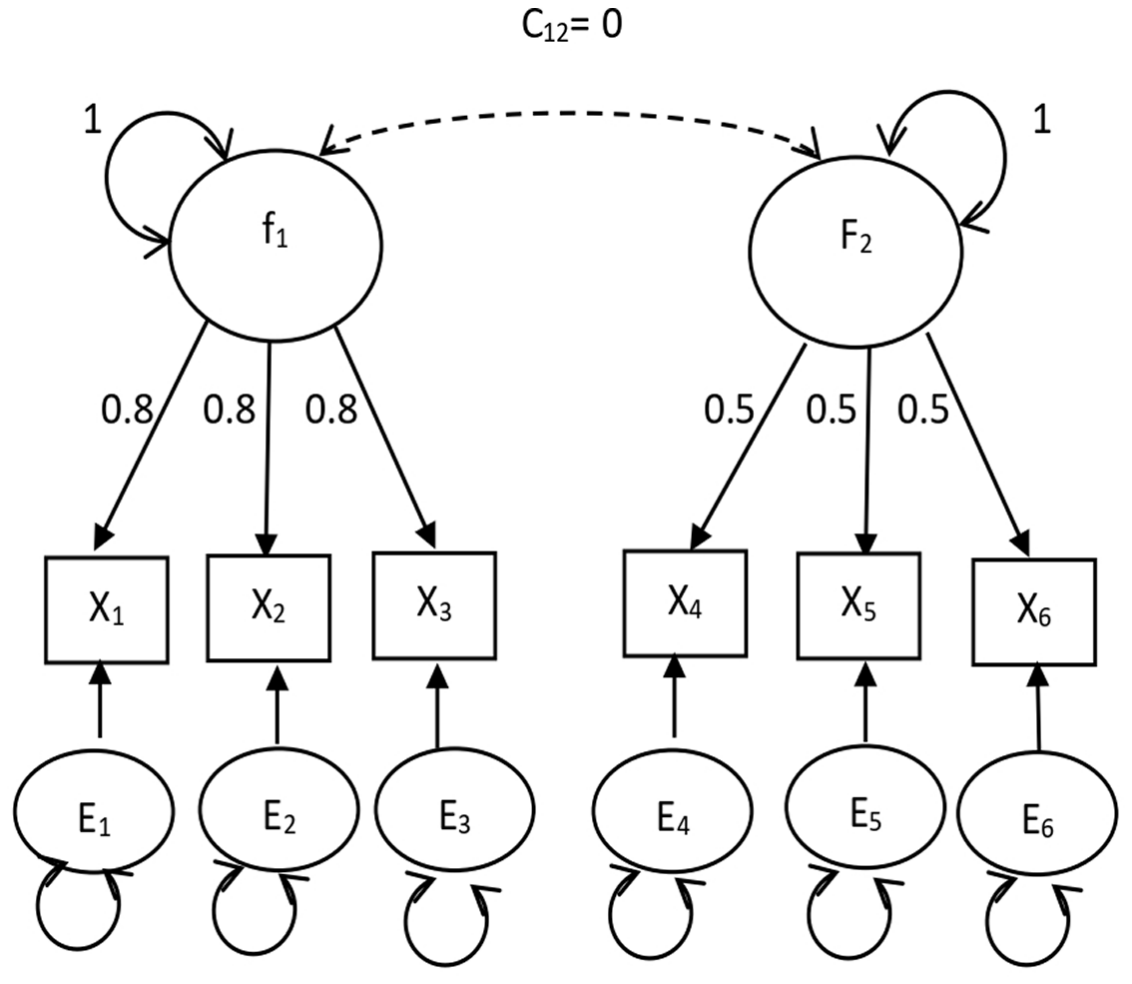
The orthogonal common factor model.

**Figure 3:**
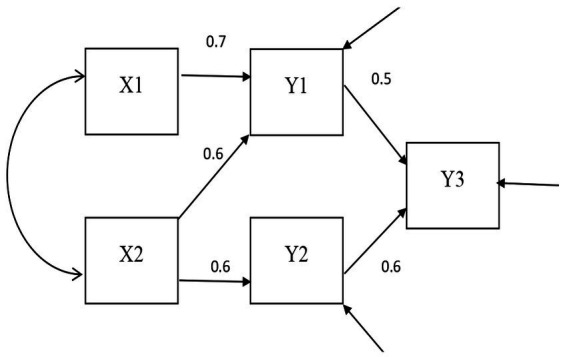
The correctly specified path model.

**Figure 4:**
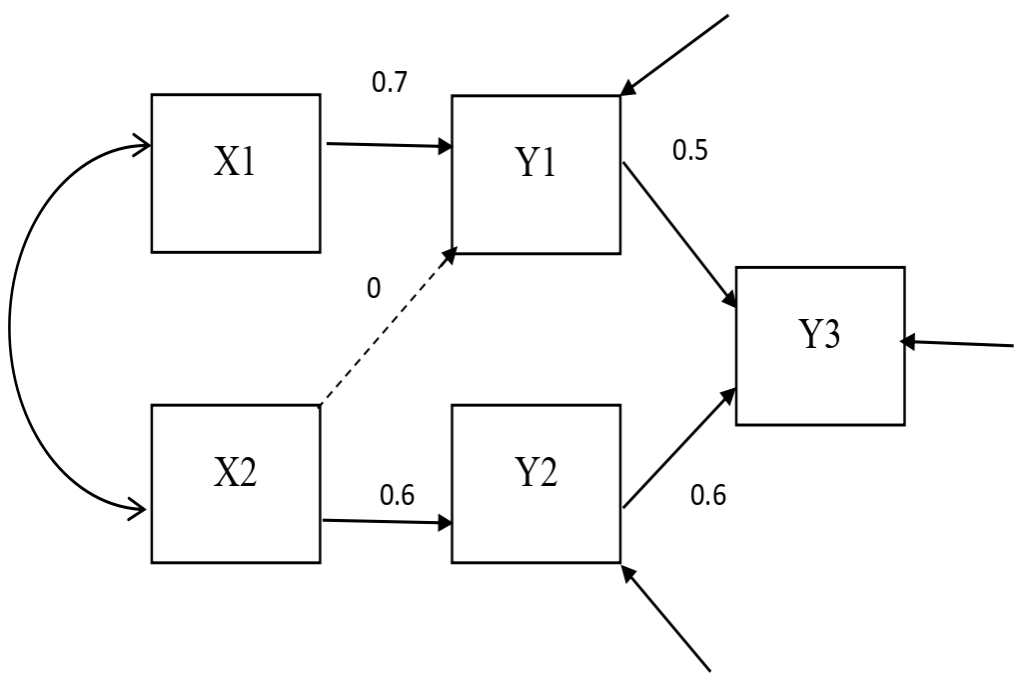
The misspecified path model.

The *DOCR*, *MD*, and *gCD* were compared. The confirmatory factor analysis models fit in Study 1 are shown in Figures 1, 2. The path analysis models fit in Study 2 are shown in Figures 3, 4. Since model misspecification can affect the identification of target cases, both correctly specified models, shown in Figures 1, 3, and misspecified models, shown in Figures 2, 4, were fit to the simulated data using the R package *lavaan* (Rosseel, 2012). The *DOCR* was calculated using basic matrix functions from the *matlib* package in R (Friendly et al., 2022). The *MD* was calculated using the *mahalanobis* function from the *stats* package that is part of base R. The *gCD* was calculated using the *genCookDist* and *explore.influence* functions from the R package *influence.SEM* (Pastore and Altoé, 2022) for both the correctly specified model and the misspecified models in both studies.

### 2.3 Implementation

Data were simulated in R v3.4.1 (R Core Team, 2017) with three different sample sizes: 200, 400, and 600. Four proportions of target cases to the number of non-target cases were applied: 0.10, 0.05, 0.02, and 0.01. The sample size and proportion of target cases were fully crossed for a factorial design with 12 conditions. The correctly specified models and misspecified models in both studies were fitted to the data using the R package *lavaan* (Rosseel, 2012). The default boxplot criterion was used to determine cases with high influence (Pastore and Altoé, 2022). The cut-off that determined multivariate outlier cases using *MD* was 12. A preliminary cut-off for *DOCR* was set at 0.01. The miss rate (*MR*) is the ratio of missed target cases to generated target cases, and the false alarm rate (*FAR*) is the ratio of flagged non-target cases to generated non-target cases. Their 95% confidence intervals were computed for each statistic for each replication using R package *psych* (Revelle, 2023). Results were averaged over 100 replications in R with confidence intervals computed using the standard error of the mean and the inverse t distribution. Averages were compared across different statistics and systematic manipulations of the conditions. Example R syntax for computing *DOCR*, as well as *gCD* and *MD*, has been provided in Appendix A. The example in Appendix A has been expanded from the package “influence.SEM” (Pastore and Altoé, 2022).

## 3 Results

The Study 1 results with the confirmatory factor analysis models are shown in Tables 1, 2, and the Study 2 results with the path analysis models are shown in Tables 3, 4. Tables 1, 3 summarize the miss rates of the three measures, *MD, DOCR*, and *gCD*, by sample size. The *DOCR* measure had the smallest miss rates compared to *MD*, *gCD-CS*, and *gCD-MS* for all sample sizes and under the four proportions of the target cases to non-target cases. In addition, the miss rate of the *DOCR* increased significantly as the sample size increased from 200 to 600 under all proportions of target cases to non-target cases. On the other hand, the miss rates of the *MD* and *gCD* remained the same when the sample size increased from 200 to 600 since their miss rates did not differ significantly with the increase in sample size for all proportions of target cases to non-target cases.

**Table 1:** Miss rates for three case detection statistics by proportions of the target to non-target cases for the CFA model.

Prop	0.1		0.05		0.02		0.01	
	*N* = 200 (180 + 20)		*N* = 200 (190 + 10)		*N* = 200 (196 + 4)		*N* = 200 (198 + 2)	
Statistic	*M*	95%CI	*M*	95%CI	*M*	95%CI	*M*	95%CI
*MD*	0.429	(0.410, 0.448)	0.340	(0.312, 0.368)	0.353	(0.308, 0.397)	0.280	(0.213, 0.347)
*DOCR*	0.024	(0.017, 0.029)	0.017	(0.010, 0.024)	0.015	(0.003, 0.027)	0.005	(−0.005, 0.015)
*gCD, CS*	0.388	(0.368, 0.408)	0.327	(0.298, 0.356)	0.315	(0.270, 0.359)	0.270	(0.208, 0.332)
*gCD, MS*	0.351	(0.329, 0.371)	0.301	(0.273, 0.329)	0.295	(0.252, 0.338)	0.230	(0.169, 0.291)
	*N* = 400 (360 + 40)		*N* = 400 (380 + 20)		*N* = 400 (392 + 8)		*N* = 400 (396 + 4)	
*MD*	0.417	(0.405, 0.429)	0.349	(0.329, 0.367)	0.303	(0.272, 0.333)	0.290	(0.245, 0.335)
*DOCR*	0.163	(0.151, 0.174)	0.116	(0.103, 0.129)	0.090	(0.071, 0.109)	0.108	(0.077, 0.138)
*gCD, CS*	0.377	(0.364, 0.391)	0.329	(0.309, 0.348)	0.288	(0.256, 0.322)	0.283	(0.238, 0.327)
*gCD, MS*	0.348	(0.334, 0.362)	0.297	(0.275, 0.319)	0.275	(0.244, 0.306)	0.275	(0.230, 0.319)
	*N* = 600 (540 + 60)		*N* = 600 (570 + 30)		*N* = 600 (588 + 12)		*N* = 600 (594 + 6)	
*MD*	0.422	(0.410, 0.433)	0.342	(0.326, 0.359)	0.331	(0.307, 0.354)	0.288	(0.253, 0.323)
*DOCR*	0.331	(0.321, 0.340)	0.259	(0.244, 0.274)	0.244	(0.220, 0.268)	0.207	(0.175, 0.238)
*gCD, CS*	0.385	(0.374, 0.395)	0.322	(0.304, 0.338)	0.318	(0.294, 0.343)	0.293	(0.257, 0.329)
*gCD, MS*	0.349	(0.339, 0.359)	0.294	(0.277, 0.311)	0.308	(0.285, 0.331)	0.275	(0.237, 0.313)

Prop, proportions of target cases to non-target cases; CI, confidence interval; MD, Malahanobis distance; CS, correctly specified model; gCD, generalized Cook’s distance; MS, misspecified model.

**Table 2:** False alarm rates for three case detection statistics by proportions of the target to non-target cases for the CFA model.

Prop	0.1		0.05		0.02		0.01	
	*N* = 200 (180 + 20)		*N* = 200 (190 + 10)		*N* = 200 (196 + 4)		*N* = 200 (198 + 2)	
Statistic	*M*	95%CI	*M*	95%CI	*M*	95%CI	*M*	95%CI
*MD*	0.024	(0.022, 0.026)	0.035	(0.033, 0.037)	0.049	(0.046, 0.052)	0.053	(0.051, 0.056)
*DOCR*	0.701	(0.692, 0.709)	0.759	(0.753, 0.765)	0.804	(0.798, 0.811)	0.820	(0.810, 0.821)
*gCD, CS*	0.059	(0.056, 0.062)	0.070	(0.066, 0.074)	0.082	(0.079, 0.086)	0.084	(0.079, 0.088)
*gCD, MS*	0.059	(0.055, 0.062)	0.070	(0.067, 0.074)	0.083	(0.079, 0.087)	0.085	(0.080, 0.089)
	*N* = 400 (360 + 40)		*N* = 400 (380 + 20)		*N* = 400 (392 + 8)		*N* = 400 (396 + 4)	
*MD*	0.023	(0.022, 0.025)	0.036	(0.034, 0.037)	0.048	(0.047, 0.049)	0.053	(0.052, 0.055)
*DOCR*	0.209	(0.205, 0.215)	0.286	(0.282, 0.292)	0.345	(0.341, 0.350)	0.367	(0.362, 0.370)
*gCD, CS*	0.058	(0.056, 0.061)	0.071	(0.068, 0.073)	0.079	(0.077, 0.082)	0.083	(0.080, 0.085)
*gCD, MS*	0.057	(0.055, 0.059)	0.072	(0.069, 0.075)	0.081	(0.079, 0.083)	0.084	(0.082, 0.087)
	*N* = 600 (540 + 60)		*N* = 600 (570 + 30)		*N* = 600 (588 + 12)		*N* = 600 (594 + 6)	
*MD*	0.023	(0.022, 0.025)	0.036	(0.035, 0.037)	0.050	(0.049, 0.051)	0.053	(0.052, 0.054)
*DOCR*	0.054	(0.052, 0.056)	0.093	(0.089, 0.095)	0.130	(0.128, 0.133)	0.142	(0.139, 0.144)
*gCD, CS*	0.057	(0.055, 0.058)	0.071	(0.069, 0.073)	0.079	(0.076, 0.080)	0.082	(0.080, 0.084)
*gCD, MS*	0.056	(0.054, 0.058)	0.072	(0.070, 0.074)	0.082	(0.079, 0.84)	0.082	(0.080, 0.084)

Prop, proportions of target cases to non-target cases; CI, confidence interval; MD, Malahanobis distance; CS, correctly specified model; gCD, generalized Cook’s distance; MS, misspecified model.

**Table 3:** Miss rates for three case detection statistics by proportions of target cases to non-target cases for the path model.

Prop	0.1		0.05		0.02		0.01	
	*N* = 200 (180 + 20)		*N* = 200 (190 + 10)		*N* = 200 (196 + 4)		*N* = 200 (198 + 2)	
Statistic	*M*	95%CI	*M*	95%CI	*M*	95%CI	*M*	95%CI
*MD*	0.225	(0.207, 0.242)	0.135	(0.114, 0.156)	0.100	(0.071, 0.129)	0.100	(0.060, 0.140)
*DOCR*	0.013	(0.008, 0.017)	0.005	(0.001, 0.009)	0.005	(0.002, 0.012)	0.000	(0.000, 0.000)
*gCD, CS*	0.103	(0.090, 0.116)	0.075	(0.058, 0.092)	0.075	(0.050, 0.099)	0.075	(0.039, 0.111)
*gCD, MS*	0.088	(0.076, 0.100)	0.060	(0.044, 0.076)	0.065	(0.041, 0.089)	0.050	(0.020, 0.080)
	*N* = 400 (360 + 40)		*N* = 400 (380 + 20)		*N* = 400 (392 + 8)		*N* = 400 (396 + 4)	
*MD*	0.229	(0.218, 0.242)	0.136	(0.121, 0.150)	0.095	(0.076, 0.114)	0.090	(0.063, 0.117)
*DOCR*	0.070	(0.063, 0.077)	0.034	(0.026, 0.041)	0.033	(0.019, 0.046)	0.025	(0.010, 0.040)
*gCD, CS*	0.101	(0.091, 0.111)	0.069	(0.058, 0.079)	0.066	(0.049, 0.083)	0.073	(0.045, 0.100)
*gCD, MS*	0.087	(0.078, 0.096)	0.054	(0.044, 0.063)	0.060	(0.044, 0.076)	0.060	(0.035, 0.086)
	*N* = 600 (540 + 60)		*N* = 600 (570 + 30)		*N* = 600 (588 + 12)		*N* = 600 (594 + 6)	
*MD*	0.232	(0.222, 0.242)	0.143	(0.131, 0.155)	0.098	(0.082, 0.113)	0.087	(0.063, 0.109)
*DOCR*	0.157	(0.147, 0.166)	0.089	(0.079, 0.100)	0.058	(0.044, 0.070)	0.033	(0.019, 0.047)
*gCD, CS*	0.105	(0.095, 0.113)	0.078	(0.069, 0.088)	0.057	(0.045, 0.068)	0.055	(0.037, 0.073)
*gCD, MS*	0.089	(0.082, 0.098)	0.068	(0.059, 0.077)	0.046	(0.035, 0.056)	0.037	(0.023, 0.050)

Prop, proportions of target cases to non-target cases; CI, confidence interval; MD, Malahanobis distance; CS, correctly specified model; gCD, generalized Cook’s distance; MS, misspecified model.

**Table 4:** False alarm rates of three case detection statistics by proportions of target cases to non-target cases for the path model.

Prop	0.1		0.05		0.02		0.01	
	*N* = 200 (180 + 20)		*N* = 200 (190 + 10)		*N* = 200 (196 + 4)		*N* = 200 (198 + 2)	
Statistic	*M*	95%CI	*M*	95%CI	*M*	95%CI	*M*	95%CI
*MD*	0.003	(0.002, 0.003)	0.007	(0.006, 0.008)	0.017	(0.015, 0.018)	0.024	(0.022, 0.026)
*DOCR*	0.421	(0.408, 0.433)	0.571	(0.560, 0.581)	0.687	(0.678, 0.697)	0.736	(0.727, 0.744)
*gCD, CS*	0.066	(0.062, 0.069)	0.086	(0.083, 0.089)	0.095	(0.092, 0.099)	0.095	(0.092, 0.99)
*gCD, MS*	0.092	(0.089, 0.095)	0.122	(0.118, 0.125)	0.137	(0.134, 0.141)	0.140	(0.136, 0.143)
	*N* = 400 (360 + 40)		*N* = 400 (380 + 20)		*N* = 400 (392 + 8)		*N* = 400 (396 + 4)	
*MD*	0.002	(0.0017, 0.003)	0.0065	(0.006, 0.007)	0.016	(0.014, 0.017)	0.021	(0.019, 0.023)
*DOCR*	0.064	(0.061, 0.068)	0.139	(0.133, 0.145)	0.237	(0.231, 0.243)	0.292	(0.285, 0.299)
*gCD, CS*	0.066	(0.064, 0.069)	0.085	(0.082, 0.088)	0.093	(0.090, 0.096)	0.094	(0.091, 0.096)
*gCD, MS*	0.092	(0.089, 0.095)	0.121	(0.119, 0.124)	0.137	(0.135, 0.139)	0.141	(0.139, 0.143)
	*N* = 600 (540 + 60)		*N* = 600 (570 + 30)		*N* = 600 (588 + 12)		*N* = 600 (594 + 6)	
*MD*	0.0018	(0.001, 0.002)	0.005	(0.0045, 0.006)	0.016	(0.014, 0.017)	0.022	(0.021, 0.023)
*DOCR*	0.011	(0.009, 0.011)	0.033	(0.031, 0.036)	0.079	(0.076, 0.082)	0.112	(0.109, 0.115)
*gCD, CS*	0.068	(0.066, 0.069)	0.083	(0.082, 0.086)	0.089	(0.087, 0.091)	0.094	(0.093, 0.097)
*gCD, MS*	0.095	(0.093, 0.097)	0.119	(0.117, 0.120)	0.134	(0.133, 0.137)	0.141	(0.139, 0.143)

Prop, proportions of target cases to non-target cases; CI, confidence interval; MD, Malahanobis distance; CS, correctly specified model; gCD, generalized Cook’s distance; MS, misspecified model.

Tables 1, 3 show that the miss rate of the *DOCR* decreased as the proportion of target cases to non-target cases decreased. The *DOCR* measure also showed the same pattern of performance under all proportions of target cases to non-target cases through all sample sizes. Similarly, the *MD* and *gCD* measures showed the same pattern of performance under all proportions of target cases to non-target cases. However, the pattern of performance for the three measures (*MD*, *DOCR*, and *gCD*) was not always statistically significant, mainly when the sample size was small.

Tables 2, 4 show the false alarm rates of the three measures, *MD, DOCR*, and *gCD*, by sample size. As these tables show, the *DOCR* measure had the highest false alarm rates compared to *MD*, *gCD–CS,* and *gCD–MS* for all sample sizes and under the four proportions of the target cases to non-target cases. Unlike the miss rate, the false alarm rate of the *DOCR* decreased as the sample size increased from 200 to 600 under all four proportions of target cases to non-target cases. In addition, the false alarm rate of the *DOCR* measure differed significantly with the increase in sample size. In other words, there was a significant decrease in the false alarm rate of the *DOCR* measure with the increase in sample size. Conversely, the false alarm rates of the *MD* and *gCD* measures did not change significantly with the increase in sample size.

Tables 2, 4 show that the false alarm rate of the *DOCR* increased as the proportion of target cases to non-target cases decreased. The *DOCR* and *MD* measures reflected the same performance pattern under all four proportions of target cases to non-target cases through all sample sizes. That is, within the same sample size, the false alarm rates of the *DOCR* and *MD* increased significantly as the proportion of the target cases to non-target cases decreased. Similarly, the *gCD* measure reflected the same performance pattern under all proportions of target cases to non-target cases. However, this performance pattern was not always statistically significant, mainly when the small sample size was relatively small.

## 4 Discussion

This study introduced the *DOCR*, a new model-free case influence measure appropriate for SEM analysis. Two simulation studies compared the performance of the *DOCR* with the performance of two other statistics that may be employed to screen cases in this context. The first was *gCD*, which is a model-based measure of case influence. Like other similar model-based case influence measures, such as likelihood distance and chi-square difference, *gCD* is sensitive to model misspecification. The greater the extent of the model misspecification, the less accurately *gCD* will identify influential cases.

The new *DOCR* statistic was also compared with the performance of *MD*. *MD* is a model-free measure. Thus, it is not sensitive to model misspecification. However, *MD* is a measure of outlying status rather than case influence. Thus, this statistic is less appropriate for detecting cases that will ultimately influence the model results.

The *DOCR* overcomes problems with both of these alternative measures employed to screen cases in SEM analysis. The *DOCR* is model-free. Thus, it is not sensitive to model misspecification. The *DOCR* is also a true case influence measure for SEM analysis, in which the model is fit to the sample covariance matrix. By detecting cases that exert a strong influence on the covariance matrix, the *DOCR* detects cases that will impact the results for the model fit to that covariance matrix.

The results of the two simulation studies suggest that more work is needed to find the optimal cut point for the *DOCR*. The *DOCR* performed better than the other measures in flagging target cases because it recorded the lowest miss rate across all conditions. However, the false alarm rate of the *DOCR* was not reasonable since it incorrectly flagged 42–80% of cases as target cases under a sample size of 200 cases. Although this percentage dropped to 10–30% when the sample size increased, it was still not satisfactory compared to other measures.

With all such measures, there is a compromise between the miss rate and the false alarm rate. Thus, the values of the false alarm rate for the *DOCR* can be made more reasonable by adjusting the cut point to yield a better balance between the miss rate and the false alarm rate. Since establishing a criterion cut point for the *DOCR* measure was outside the scope of this study, it is recommended that future studies establish an optimal cut point criterion for this measure.

The results of the two simulation studies also suggest that the *DOCR* is sensitive to sample size. The *DOCR*’s miss rate increased, and the false alarm rate decreased significantly with an increase in sample size, while the miss rate and false alarm rate of *MD* and *gCD* remained the same. This finding was consistent with previous studies. Previous studies have noted how sample size may affect the performance of case influence measures because the influence of the individual case is weighted by the inverse of the sample size (Pek and MacCallum, 2011). Therefore, a large influence is expected from individual cases in small samples. The findings of this study were consistent with studies that showed the performance of some measures, such as chi-square, that were extremely sensitive to sample size (Boomsma, 1982; Fan et al., 1999). Future studies should investigate methods for reducing the sensitivity of the *DOCR* to sample size.

Given these two limitations, practitioners are recommended not to use the *DOCR* measure with overly small sample sizes (i.e., *N* ≤ 200) or overly large sample sizes (i.e., N>600 ). Instead, practitioners should use the range of sample sizes recommended for SEM studies (Kline, 2016) to obtain the best performance of the *DOCR* measure. Care should be exercised in investigating the cases that are flagged, considering that some of the influential cases identified may be due to sampling variability alone. However, used within these guidelines, the *DOCR* shows promise as a model-free case influence measure appropriate for SEM analysis due to its ability to overcome the limitations of existing measures. Example R syntax for computing *DOCR* has been provided in the Appendix.

## Data availability statement

The raw data supporting the conclusions of this article will be made available by the authors, without undue reservation.

## Author contributions

FJ and JK designed the study and created the routine. FJ contributed to the write-up of the manuscript, the R code for the DOCR and other indices, the analysis of the data, and summarizing the results. JK contributed to the write-up of the manuscript and to the improvement of all sections of this manuscript. All authors contributed to the article and approved the submitted version.
