# Supplementary material for: Model-free measurement of case influence in structural equation modeling
Source: Front Psychol. 2024 Feb 6;14:1245863. doi: 10.3389/fpsyg.2023.1245863 (PMC10878323; doi:10.3389/fpsyg.2023.1245863)
Supplement: Supplementary file 1 [file Data_Sheet_1.pdf]

APPENDIX A

```
# install package needed for inv function
if (!require('matlib')) install.packages('matlib')
# define newfunction to compute DOCR
newfunction <- function (data, ...)
{ DOCR <- NULL
# compute full data covariance matrix
cov0 <- cov(data,...)
DOCR <- sapply(1:nrow(data), function(i) {
  # compute delete one covariance matrix
  covi <- cov(data[-i, ], ...)
  # compute standardized residuals
  stdizd.res <- (cov0 - covi)%*%matlib::inv(cov0)
  # eliminate duplicate entries above main diagonal
  stdizd.res[upper.tri(stdizd.res)] <- 0
  # vectorize the stdizd residuals
  vec.res <- as.vector(stdizd.res)
  # sum of squared stdizd residuals
  SS.res <- t(vec.res)%*%vec.res
  # divide by number of elements in cov matrix, multiply 1000
  nu <- nrow(cov0)
  p <- nu*(nu+1)/2
  (SS.res/p)*1000})
return(DOCR)}
# install package needed for PDII data and generalized Cook's distance
if (!require('influence.SEM')) install.packages('influence.SEM'); library(influence.SEM)
data(PDII)
# calculate DOCR
```

```
DOCR<- newfunction(PDII)
# calculate mahalanobis distance
MD <- mahalanobis(PDII, colMeans(PDII), cov(PDII))
# The following example is from the influence.SEM documentation (Pastore & Altoe', 2022)
citation('influence.SEM')
# define model needed for generalized Cook's distance
model <- "
F1 =~ x1+x2+x3
F2 =~ y1+y2+y3+y4
F3 =~ y5+y6+y7+y8"
# calculate generalized Cook's distance
gCD <- influence.SEM::genCookDist(model,data=PDII,std.lv=TRUE)
extract <- influence.SEM::explore.influence(gCD,cook=TRUE)
extract
```
